# Editorial: Education in obstetrics and gynecology

**DOI:** 10.3389/fmed.2024.1490673

**Published:** 2024-11-05

**Authors:** Florian Recker, Fedde Scheele

**Affiliations:** ^1^Department of Obstetrics and Prenatal Medicine, University Hospital Bonn, Bonn, Germany; ^2^Athena Institute, Faculty of Science, Vrije Universiteit Amsterdam, Amsterdam, Netherlands

**Keywords:** medical education, obstetrics, gynecology, continuing medical education, training

Medical education in obstetrics and gynecology (OB-GYN) has undergone significant transformations to reach its current state, with the field adapting to advancements in technology, evolving patient needs, and a growing emphasis on multidisciplinary approaches to healthcare. This dynamic field, crucial to women's health, now requires a more comprehensive and integrative educational framework to prepare future clinicians for the complex challenges they will face in their practice. High-quality education and training of medical professionals are fundamental pillars in ensuring the health and wellbeing of global populations. In particular, OB-GYN requires continuous advancements in educational strategies to address the unique challenges presented by a globalized and interconnected world. This Research Topic seeks to explore the multifaceted aspects of medical education within OB-GYN, spanning from undergraduate studies to postgraduate education, continuing medical education (CME), and beyond.

The journey to becoming an OB-GYN specialist begins with a robust undergraduate medical education. Here, it is imperative to develop a curriculum that not only covers the essential medical knowledge but also fosters critical thinking, empathy, and self-reflective skills (1). Modern medical schools are increasingly adopting integrated curricula that blend basic sciences with clinical practice early on (2). This approach helps students contextualize their learning and understand the relevance of theoretical knowledge in real-world scenarios. Innovative teaching methods, such as problem-based learning (PBL) and team-based learning (TBL), have shown significant promise in enhancing student engagement and retention of knowledge (3). These methodologies encourage collaborative learning, critical analysis, and the application of knowledge to practical problems, preparing students for the dynamic and often unpredictable nature of clinical practice in OB-GYN (4).

In the context of obstetrics and gynecology education, competency-based ultrasound education has emerged as a pivotal component of training programs (Weimer et al.). This approach ensures that learners develop the essential skills and knowledge required for effective ultrasound use, regardless of the time it takes to achieve these competencies. Rather than progressing through a fixed curriculum, students advance based on their demonstrated proficiency in various aspects of ultrasound, from image acquisition and interpretation to the integration of findings into clinical decision-making (5). Simulation-based training plays a significant role, providing learners with opportunities to practice and refine their skills in a controlled environment until they reach a level of mastery. This method not only enhances technical abilities but also promotes critical thinking and clinical judgment, ensuring that graduates are fully prepared to utilize ultrasound as a diagnostic and therapeutic tool in their practice. By focusing on competency rather than time spent in training, this educational model better equips future obstetricians and gynecologists to meet the demands of modern healthcare.

Postgraduate education, particularly residency training, is where the foundational knowledge acquired during undergraduate studies is honed into specialized clinical skills (Plöger et al.). Residency programs for OB-GYN must be rigorous, inclusive, and comprehensive, ensuring that trainees are well-equipped to handle the complexities of the field. Standardized accreditation and validation processes are crucial to maintaining the quality and consistency of these programs globally. Simulation-based training has become a cornerstone in residency programs, providing a safe and controlled environment for residents to practice and refine their skills. High-fidelity simulations, including the use of virtual reality (VR) and augmented reality (AR), offer immersive experiences that mimic real-life clinical scenarios. These technologies not only enhance technical skills but also improve decision-making, teamwork, and communication—essential components of effective clinical practice.

The rapid pace of medical advancements necessitates a commitment to lifelong learning for OB-GYN professionals. Continuing medical education (CME) ensures that practitioners remain up to date with the latest developments, techniques, and best practices in the field. CME programs must be flexible, accessible, and relevant, catering to the diverse needs of healthcare professionals across different stages of their careers. Digital platforms have revolutionized CME, offering online courses, webinars, and virtual conferences that enable professionals to learn at their own pace and convenience (6). Additionally, mobile applications and e-learning modules provide on-the-go access to educational resources, allowing clinicians to seamlessly integrate learning into their daily routines.

The digitalization of medical education has opened up new avenues for teaching and training. VR and AR are transforming the way medical students and professionals learn, providing immersive and interactive experiences that enhance understanding and retention (7). These technologies can simulate complex OB-GYN procedures, offering hands-on practice without the risks associated with real-life interventions. Simulation centers equipped with high-fidelity mannequins and task trainers allow for the practice of intricate surgical techniques, obstetric emergencies, and patient management scenarios. These controlled environments enable learners to make mistakes and learn from them, building confidence and competence.

Healthcare is inherently multidisciplinary, and the education of OB-GYN professionals must reflect this reality. Inter-professional training, where medical students, nursing students, and other healthcare trainees learn together, promotes a collaborative approach to patient care. This model fosters mutual respect, understanding, and communication among different healthcare professionals, ultimately improving patient outcomes. Transdisciplinary approaches, which incorporate non-medical education such as ethics, communication, and leadership training, further enrich the learning experience. These elements are crucial in developing well-rounded healthcare professionals who are not only skilled clinicians but also empathetic leaders and effective communicators.

Qualitative and quantitative research in medical education is essential for continuous improvement and innovation. Studies that evaluate the effectiveness of different teaching methods, curricular designs, and training tools provide valuable insights that inform educational practices. Research also helps identify gaps and areas for improvement, ensuring that medical education evolves to meet the changing needs of society.

The education of healthcare professionals in Obstetrics and Gynecology is a dynamic and evolving field that requires a multifaceted approach. From undergraduate education to postgraduate training and continuing medical education, each stage plays a critical role in developing competent, compassionate, and resilient practitioners. The integration of innovative teaching tools, inter-professional education, and rigorous research is essential to meet the challenges of a globalized world and ensure the wellbeing of global populations. As we look to the future, it is imperative that medical education remains inclusive, collaborative, and forward-thinking. By embracing these principles, we can train the next generation of OB-GYN professionals who are not only skilled clinicians but also advocates for health and wellbeing in their communities and beyond. This Research Topic aims to shed light on these important Research Topics, providing a platform for sharing knowledge, experiences, and best practices in the education of OB-GYN professionals.

The 17 contributions for this Research Topic concerning education in obstetrics and gynecology can be classified into different sections. For that purpose, the article by Frenk et al. in the Lancet, is still valuable (8). He categorized learning into three areas: specific skills needed to become a medical expert, general competencies required for professionalism, and transformative, future-focused learning to develop as a change agent. Eleven articles concern program evaluation, of which two describe checklists to assess either a skill or a generic competency, i.e., teamwork, and two articles describe program evaluation focused on generic competencies. The third class of articles are those concerning the future of education. They concern the changing demographics of our workforce and the need for culture changes, as artificial intelligence and digital tools are introduced in both our learning and care provision systems. Learning for the future in a fast-changing world also demands elements of transformative learning to foster change-agents in the health sector. The item of transformative learning is still in its infancy and needs far more academic attention. Hopefully, in a next Research Topic of this journal, the connection between health system transformation and education may attract more articles from this emerging field. Finally, an article from Ethiopia reminds us that sociological, economic, and cultural features of society are extremely relevant for addressing health issues. Learning about public health, with a broad view on society and its influence on health care, is essential for the quality and efficiency of future health services.

The landscape of medical education in obstetrics and gynecology is characterized by technological innovation, a shift toward competency-based learning, interdisciplinary collaboration, and a strong commitment to global health and equity. These advancements ensure that future OB-GYN practitioners are well-prepared to meet the complex demands of their field, providing high-quality care to women across diverse settings. As the field continues to evolve, so too will the educational strategies, maintaining a focus on excellence and adaptability in the face of new challenges.

We hope that both obstetrician-gynecologists and teachers will enjoy this Research Topic and get engaged in optimal training for the benefit of global health.
